# COVID-19 in patients with autoimmune diseases: characteristics and outcomes in a multinational network of cohorts across three countries

**DOI:** 10.1093/rheumatology/keab250

**Published:** 2021-03-16

**Authors:** Eng Hooi Tan, Anthony G Sena, Albert Prats-Uribe, Seng Chan You, Waheed-Ul-Rahman Ahmed, Kristin Kostka, Christian Reich, Scott L Duvall, Kristine E Lynch, Michael E Matheny, Talita Duarte-Salles, Sergio Fernandez Bertolin, George Hripcsak, Karthik Natarajan, Thomas Falconer, Matthew Spotnitz, Anna Ostropolets, Clair Blacketer, Thamir M Alshammari, Heba Alghoul, Osaid Alser, Jennifer C E Lane, Dalia M Dawoud, Karishma Shah, Yue Yang, Lin Zhang, Carlos Areia, Asieh Golozar, Martina Recalde, Paula Casajust, Jitendra Jonnagaddala, Vignesh Subbian, David Vizcaya, Lana Y H Lai, Fredrik Nyberg, Daniel R Morales, Jose D Posada, Nigam H Shah, Mengchun Gong, Arani Vivekanantham, Aaron Abend, Evan P Minty, Marc Suchard, Peter Rijnbeek, Patrick B Ryan, Daniel Prieto-Alhambra

**Affiliations:** 1 Centre for Statistics in Medicine, Nuffield Department of Orthopaedics, Rheumatology, and Musculoskeletal Sciences, University of Oxford, OX3 7LD, UK; 2 Janssen Research and Development, Titusville, NJ USA; 3 Department of Medical Informatics, Erasmus University Medical Center, Rotterdam, The Netherlands; 4 Department of Biomedical Informatics, Ajou University School of Medicine, Suwon, Korea; 5 Nuffield Department of Orthopaedics, Rheumatology, and Musculoskeletal Sciences, University of Oxford, Botnar Research Centre, Oxford, OX3, 7LD, UK; 6 College of Medicine and Health, University of Exeter, St Luke’s, 2LU, USA; 7 Real World Solutions, IQVIA, Cambridge, MA, USA; 8 VA Informatics and Computing Infrastructure, VA Salt Lake City Health Care System, UT, USA; 9 Department of Internal Medicine, University of Utah School of Medicine, Salt Lake City, UT, USA; 10 Tennessee Valley Healthcare System, Veterans Affairs Medical Center, Nashville, TN, USA; 11 Department of Biomedical Informatics, Vanderbilt University Medical Center, Nashville, TN, USA; 12 Fundació Institut Universitari per a la recerca a l'Atenció Primària de Salut Jordi Gol i Gurina (IDIAPJGol), Barcelona, Spain; 13 Department of Biomedical Informatics, Columbia University, New York, NY, USA; 14 New York-Presbyterian Hospital, New York, NY, USA; 15 Medication Safety Research Chair, King Saud University, Riyadh, Saudi Arabia; 16 Faculty of Medicine, Islamic University of Gaza, Palestine; 17 Massachusetts General Hospital, Harvard Medical School, Boston, 02114, MA, USA; 18 Faculty of Pharmacy, Cairo University, Cairo, Egypt; 19 Digital China Health Technologies Co., LTD, Beijing 100085, China; 20 School of Population Medicine and Public Health, Chinese Academy of Medical Science & Peking Union Medical College, Beijing 100730, China; 21 Melbourne School of Population and Global Health, The University of Melbourne, Victoria 3015, Australia; 22 Nuffield Department of Clinical Neurosciences, University of Oxford, OX3 9DU, UK; 23 Regeneron Pharmaceuticals, NY, USA; 24 Department of Epidemiology, Johns Hopkins Bloomberg School of Public Health,, Baltimore, MD, USA; 25 Universitat Autonoma de Barcelona, Bellaterra, Spain; 26 Real-World Evidence, Trial Form Support, Barcelona, Spain; 27 School of Public Health and Community Medicine, UNSW Sydney, Australia; 28 College of Engineering, The University of Arizona Tucson, Arizona, USA; 29 Bayer Pharmaceuticals, Sant Joan Despi, Barcelona, Spain; 30 School of Medical Sciences, University of Manchester, UK; 31 School of Public Health and Community Medicine, Institute of Medicine, Sahlgrenska Academy, University of Gothenburg, Gothenburg, Sweden; 32 Division of Population Health Sciences, University of Dundee, Dundee, Scotland, UK; 33 Stanford Center for Biomedical Informatics Research, Department of Medicine, School of Medicine, Stanford University, Stanford, CA, USA; 34 Health Management Institute, Southern Medical University, Guangzhou, China; 35 Autoimmune Registry Inc., Guilford, CT 06437, USA; 36 O’Brien School for Public Health, Faculty of Medicine, University of Calgary, Calgary, Alberta, T2N, 1N4, Canada; 37 Department of Biostatistics, UCLA Fielding School of Public Health, University of California, Los Angeles, CA, USA

**Keywords:** COVID-19, autoimmune condition, mortality, hospitalization, open science, Observational Health Data Sciences and Informatics (OHDSI), Observational Medical Outcomes Partnership (OMOP)

## Abstract

**Objective:**

Patients with autoimmune diseases were advised to shield to avoid coronavirus disease 2019 (COVID-19), but information on their prognosis is lacking. We characterized 30-day outcomes and mortality after hospitalization with COVID-19 among patients with prevalent autoimmune diseases, and compared outcomes after hospital admissions among similar patients with seasonal influenza.

**Methods:**

A multinational network cohort study was conducted using electronic health records data from Columbia University Irving Medical Center [USA, Optum (USA), Department of Veterans Affairs (USA), Information System for Research in Primary Care-Hospitalization Linked Data (Spain) and claims data from IQVIA Open Claims (USA) and Health Insurance and Review Assessment (South Korea). All patients with prevalent autoimmune diseases, diagnosed and/or hospitalized between January and June 2020 with COVID-19, and similar patients hospitalized with influenza in 2017–18 were included. Outcomes were death and complications within 30 days of hospitalization.

**Results:**

We studied 133 589 patients diagnosed and 48 418 hospitalized with COVID-19 with prevalent autoimmune diseases. Most patients were female, aged ≥50 years with previous comorbidities. The prevalence of hypertension (45.5–93.2%), chronic kidney disease (14.0–52.7%) and heart disease (29.0–83.8%) was higher in hospitalized *vs* diagnosed patients with COVID-19. Compared with 70 660 hospitalized with influenza, those admitted with COVID-19 had more respiratory complications including pneumonia and acute respiratory distress syndrome, and higher 30-day mortality (2.2–4.3% *vs* 6.32–24.6%).

**Conclusion:**

Compared with influenza, COVID-19 is a more severe disease, leading to more complications and higher mortality.

**Rheumatology key messages**

- Most patients with autoimmune diseases hospitalized for COVID-19 were women, older, and had previous comorbidities.
- There is a higher prevalence of hypertension, chronic kidney disease, heart disease in patients with autoimmune diseases hospitalized with COVID-19.
- Patients with autoimmune diseases, hospitalized with COVID-19, had worse outcomes and 30-day mortality compared to influenza.

## Introduction

Millions of people have died from coronavirus disease 2019 (COVID-19) globally [1]. There is concern that patients with autoimmune diseases are at an increased risk of infection and complications, exacerbated by the nature of their disease and/or the use of immunosuppressive therapies [2]. In addition, systemic inflammation is present in many autoimmune diseases [3], leading to an increased risk of cardiovascular [3–5] and thromboembolic disease [6–8], which have also been recently reported to be associated with COVID-19. In patients infected with COVID-19, worse outcomes such as hospitalization, requiring intensive services and death may be associated with a pro-inflammatory cytokine storm [9–11]. Currently identified general risk factors for COVID-19 hospitalization include systemic autoimmune diseases among other comorbidities [12, 13].

As having autoimmune diseases is a recognized risk factor for COVID-19 related complications [2], public health authorities around the world have advised mitigation strategies for those at risk. In the absence of a vaccine and a scarcity of proven therapeutic options, non-pharmacological measures such as shielding, case isolation, strict hand hygiene and social distancing are key measures to protect this vulnerable group of patients [14, 15]. Thus far, characterization studies about COVID-19 infection in people with autoimmune conditions have been limited in sample size and mostly region-specific [12, 13, 16–19]. As such, COVID-19 outcomes among people with autoimmune conditions remain poorly understood.

With the ongoing threat of COVID-19, clinical understanding of the characteristics and prognosis of patients with autoimmune conditions will facilitate the management of care for this group of patients. Given the paucity of evidence, our study aimed to describe the patients’ socio-demographics, comorbidities and 30-day complications and mortality among patients with prevalent autoimmune conditions hospitalized with COVID-19 across North America, Europe and Asia. In addition, we compared their health outcomes and mortality with those seen in patients with autoimmune diseases hospitalized with seasonal influenza in the previous years.

## Methods

### Study design and data sources

We conducted a multinational network retrospective cohort study as part of the Characterizing Health Associated Risks, and Your Baseline Disease In Severe acute respiratory syndrome coronavirus 2 (SARS-CoV-2) (CHARYBDIS) protocol [20]. At time of publication, there were 18 databases contributing to CHARYBDIS. All data were standardized to the Observational Medical Outcomes Partnership (OMOP) Common Data Model [21], which allowed a federated network analysis without sharing patient-level data. In this study, we selected databases with more than 140 patients meeting our inclusion criteria to secure sufficient precision with a confidence interval width of ± 5% in the study of the prevalence of a previous condition or 30-day risk of an outcome affecting 10% of the study population.We included six data sources from three countries, namely the USA, Spain and South Korea, including hospital out- and inpatient electronic health records (EHRs) from Columbia University Irving Medical Center (CUIMC) USA, Optum (Optum EHR) (USA), Department of Veterans Affairs (VA-OMOP) (USA), primary care EHR linked to hospital admissions data from the Information System for Research in Primary Care-Hospitalization Linked Data (SIDIAP-H) (Spain) [22], and health claims from IQVIA Open Claims (USA) and Health Insurance and Review Assessment (HIRA) (South Korea) [23]. A flowchart of the databases included and excluded from those available in the network is shown in Supplementary Fig. S1, available at *Rheumatology* online and a detailed description of the included databases can be found in Supplementary Appendix 1, available at *Rheumatology* online.

### Study participants and follow-up

For the COVID-19 cohort, all patients diagnosed and/or hospitalized between January and June 2020 with a clinical or laboratory-confirmed diagnosis of COVID-19 and with one or more prevalent autoimmune diseases were included. For the influenza cohort, all patients diagnosed and/or hospitalized between September 2017 and April 2018 with a clinical or laboratory-confirmed diagnosis of influenza and with one or more prevalent autoimmune diseases were included. The index date (i.e. start time of the cohort) was the date of diagnosis or of hospital admission, respectively. All participants were required to have at least 365 days of observational data prior to the index date. Prevalent autoimmune condition was defined as patients having any of the following conditions captured in the data source, any time prior to the index date: Type 1 diabetes mellitus, RA, psoriasis, PsA, multiple sclerosis, SLE, Addison’s disease, Graves’ disease, SS, Hashimoto thyroiditis, myasthenia gravis, vasculitis, pernicious anaemia, coeliac disease, scleroderma, sarcoidosis, ulcerative colitis or Crohn’s disease.

Participants were followed up for the identification of study outcomes from the index date until the earliest of death, end of the study (June 2020), 30 days after index or last date of data availability.

### Baseline characteristics

Socio-demographics (age and sex) at index date were extracted, together with comorbidities and medicines used as recorded in the 365 days prior to the index date. All features recorded in the analysed databases were extracted, and are fully reported together with study outcomes (see below) in an aggregated form in an interactive web application (https://data.ohdsi.org/Covid19CharacterizationCharybdis/).

### Study outcomes

For the diagnosed patients, we identified hospitalization episodes in the 30 days after the index date. For the hospitalized patients, we identified the following outcomes in the 30 days after the index date: acute myocardial infarction, cardiac arrhythmia, heart failure, stroke, venous thromboembolism, sepsis, acute respiratory distress syndrome (ARDS), pneumonia, acute kidney injury and mortality. The outcomes were defined using code sets based on Systematized Nomenclature of Medicine, Current Procedural Terminology, 4th Edition or International Classification of Diseases 9th edition (ICD-9)/10th edition (ICD-10) disease or procedure codes. Outcomes were not reported for the SIDIAP-H database as these were all hospital-based diagnoses and therefore highly incomplete in primary care EHR data. Mortality will only be reported for the following data sources which have good quality and complete data: CUIMC, HIRA, SIDIAP-H and VA-OMOP.

### Data characterization and analysis

A common analytical package was developed based on the Observational Health Data Sciences and Informatics (OHDSI) Methods library (available at https://github.com/ohdsi-studies/Covid19CharacterizationCharybdis) and run locally in each database in a distributed network fashion [24, 25]. Results were extracted on 3 October 2020, and are constantly updated with new data in the web application.

We reported patient socio-demographics, comorbidities and commonly used medications in the 365 days before index date. The index date for the diagnosed cohort is the earlier of the date of clinical diagnosis or laboratory confirmed diagnosis using SARS-COV2 test; whereas the index date for the hospitalized cohort is the date of admission. We calculated the absolute standardized mean difference (ASMD) for patient characteristics between the diagnosed and hospitalized with COVID-19 cohorts. The ASMD is calculated as a difference in prevalence between the diagnosed and hospitalized groups, divided by the difference in standard deviation of the prevalence of these two groups. Guidelines indicate that ASMD >0.1 represent that the prevalence in the two groups are different from one another [26]. We calculated the proportion of hospitalization among diagnosed patients and the proportion of hospitalized patients having severe outcomes (acute myocardial infarction, cardiac arrhythmia, heart failure, stroke, venous thromboembolism, sepsis, ARDS, pneumonia, acute kidney injury and mortality) within 30 days post index date. We compared outcomes and mortality to patients with a history of autoimmune diseases hospitalized with influenza in the previous 2017–18 season. This study was descriptive in nature, and no causal inference was intended. Multivariable regression or adjustment for confounding was therefore considered beyond the purpose and scope of our study, and not included in our study protocol. All analyses were performed and visualized using R (version 4.0.2) [27].

### Patient and public involvement statement

No patients or public were involved in the design, execution or dissemination of this study.

## Ethics approval

All the data partners received Institutional Review Board (IRB) approval or exemption. The use of VA data was reviewed by the Department of Veterans Affairs Central IRB and was determined to meet the criteria for exemption under Exemption Category 4(3) and approved the request for Waiver of Health Insurance Portability and Accountability Act (HIPAA) Authorization. The research was approved by the Columbia University Institutional Review Board as an OHDSI network study. The IRB number for use of HIRA data was AJIB-MED-EXP-20–065. SIDIAP analysis was approved by the Clinical Research Ethics Committee of the IDIAPJGol (project code: 20/070-PCV). Other databases used (IQVIA Open Claims and Optum EHR) are commercially available, syndicated data assets that are licensed by contributing authors for observational research. These assets are de-identified commercially available data products that could be purchased and licensed by any researcher. The collection and de-identification of these data assets is a process that is commercial intellectual property and not privileged to the data licensees and the co-authors on this study. Licensees of these data have signed Data Use Agreements with the data vendors which detail the usage protocols for running retrospective research on these databases. All analyses performed in this study were in accordance with Data Use Agreement terms as specified by the data owners. As these data are deemed commercial assets, there is no IRB applicable to the usage and dissemination of these result sets or required registration of the protocol with additional ethics oversight. Compliance with Data Use Agreement terms, which stipulate how these data can be used and for what purpose, is sufficient for these commercial entities. Further inquiry related to the governance oversight of these assets can be made with the respective commercial entities: IQVIA (iqvia.com) and Optum (optum.com). At no point in the course of this study were the authors of this study exposed to identified patient-level data. All result sets represent aggregate, de-identified data that are represented at a minimum cell size of >5 to reduce potential for re-identification.

## Results

We included 133 589 patients (129 221 from USA, 3553 from Spain and 815 from South Korea) with prevalent autoimmune diseases and a clinical diagnosis of COVID-19 or a positive SARS-CoV-2 test (Table 1). The claims databases (HIRA and IQVIA Open Claims) did not have information on laboratory confirmed results. The proportions of laboratory-confirmed COVID-19 cases ranged from 37.1 to 64.0% for the diagnosed cohort and 46.2 to 90.3% for the hospitalized cohort. Patients were mainly female in CUIMC (63.8%), HIRA (63.4%), IQVIA Open Claims (60.5%), Optum EHR (65.9%) and SIDIAP-H (62.0%) but were predominantly male in VA-OMOP (88.3%), as expected given the population based on military veterans. The majority of cases were aged ≥50 years. Among these patients with autoimmune diseases who developed COVID-19, the most prevalent autoimmune conditions were psoriasis (3.5–27.9%), RA (4.0–18.9%) and vasculitis (3.3–17.5%). The most prevalent comorbidities were hypertension (42.0–85.2%), heart disease (29.2–71.1%), Type 2 diabetes (21.7–63.3%) and hyperlipidaemia (22.7–59.2%). Except for HIRA, in which obesity recording rate is low, obesity was a frequently diagnosed comorbidity in all other databases (44.4–63.1%). The most frequently prescribed medications in the year prior to COVID-19 diagnosis across all databases were systemic antibiotics (48.2–84.2%), drugs used for gastro-oesophageal reflux disease (GERD) (39.1–80.6%) and NSAIDs (31.3–77.5%).

**Table 1:** Baseline characteristics of study participants diagnosed with COVID-19 and had prevalent autoimmune diseases, stratified by data source

Covariate	CUIMC (USA) (*n* = 1363)	HIRA (South Korea) (*n* = 815)	IQVIA open claims (USA) (*n* = 104874)	Optum EHR (USA) (*n* = 12897)	SIDIAP-H (Spain) (*n* = 3553)	VA-OMOP (USA) (*n* = 10087)
Female	63.8	63.4	60.5	65.9	62.0	11.7
Age group						
≤19	0.7	0.7	0.8	1.7	1.3	0
20–29	3.0	8.7	2.6	6.0	4.4	0.6
30–39	8.7	4.5	5.6	9.8	10.2	4.5
40–49	11.9	9.9	9.9	14.8	18.0	7.0
50–59	16.7	26.6	17.9	21.9	17.3	15.7
60–69	21.4	21.8	22.1	21.9	14.5	26.2
70–79	17.9	15.5	19.9	13.8	13.1	34.2
80–89	14.3	9.9	21.2	10.1	14.4	8.6
90–99	4.3	1.8	0.0	0.0	6.8	3.2
Autoimmune disease in the year prior to index date^a^						
Type 1 diabetes mellitus	3.4	1.5	5.8	6.0	5.0	4.4
RA	4.0	18.9	4.8	8.7	4.1	4.7
Psoriasis	3.7	8.2	3.5	7.4	27.9	7.1
PsA	0.8	0.7	0.8	2.4	2.2	1.5
Multiple sclerosis	2.1	<0.6	2.2	3.3	2.2	1.9
SLE	3.4	1.7	1.9	3.6	2.3	1.1
Graves’ disease	0.0	0.0	0.0	0.1	0.0	0.0
Hashimoto thyroiditis	0.0	0.0	0.0	2.1	0.0	0.0
Myasthenia gravis	0.5	<0.6	0.4	0.6	1.0	0.6
Vasculitis	4.2	14.4	4.0	5.7	17.5	3.3
Pernicious anaemia	0.0	0.0	0.0	0.5	0.0	0.0
Coeliac disease	0.9	<0.6	0.5	1.6	5.1	0.7
Scleroderma	0.6	<0.6	0.2	0.4	0.8	0.2
Sarcoidosis	2.7	0.0	1.0	2.1	1.1	2.6
Ulcerative colitis	1.9	<0.6	1.3	2.5	4.1	2.6
Crohn's disease	2.3	<0.6	1.2	3.0	2.9	1.6
Comorbidities in the year prior to index date						
Hyperlipidaemia	34.2	56.4	40.1	40.9	22.7	59.2
Asthma	27.0	28.0	23.3	22.7	8.3	15.1
CKD	33.0	14.2	35.6	27.8	15.8	38.1
COPD	18.8	3.4	24.1	16.7	27.4	40.2
Dementia	12.6	12.3	19.0	5.6	7.3	13.4
Heart disease	66.9	29.2	71.1	48.0	32.5	70.0
HIV	3.1	NA	2.1	0.7	0.4	2.0
Hypertension	73.5	45.6	81.7	60.4	42.0	85.2
Cancer	32.4	8.5	24.9	25.2	15.6	32.3
Obesity	59.4	NA	44.4	63.1	45.5	63.0
Type 2 diabetes mellitus	48.1	46.3	62.6	36.7	21.7	63.3
Cerebrovascular disease	6.7	7.7	8.4	4.7	3.3	6.9
Chronic liver disease	2.8	10.2	2.0	2.5	2.7	6.4
Pregnancy	2.7	2.0	1.1	2.3	0.9	0.1
Venous thromboembolism	7.4	3.4	6.1	4.9	11.5	6.4
Drug utilization in the year prior to index date						
Agents acting on the renin–angiotensin system	29.3	29.6	30.6	31.1	31.6	47.7
Antibacterials	48.2	84.2	54.2	49.8	51.7	54.8
Antidepressants	23.3	21.2	23.7	31.4	30.6	45.8
Antineoplastic and immunomodulating agents	22.9	10.2	15.6	21.8	13.3	21.3
Antithrombotics	38.0	47.1	23.5	36.2	31.9	52.7
Beta blockers	29.2	17.3	26.8	28.0	19.2	44.7
Calcium channel blockers	26.7	25.5	21.4	19.6	14.5	33.8
Corticosteroids	36.4	72.3	38.4	45.0	39.6	43.9
Diuretics	29.3	17.5	26.2	27.1	29.0	39.3
Drugs for obstructive airway diseases	31.0	30.2	39.1	41.2	28.8	55.1
GERD	39.1	80.6	51.4	39.2	50.7	72.8
PPI	28.9	50.2	24.4	31.8	49.9	44.6
Lipid modifying agents	37.0	34.5	35.2	36.1	26.8	64.4
NSAID	31.3	77.5	51.2	35.4	36.8	75.5
Opioids	24.5	82.1	24.4	29.0	21.8	30.4

Figures are presented in percentages; the figures preceded with < denote less than five people in that category. Age groups are collapsed from 5-year age bands; percentages are not summed if one of the age categories had less than five people.

a These are not mutually exclusive, and classification is based on recent (1 year prior) records.

CUIMC: Columbia University Irving Medical Center; HIRA: Health Insurance Review and Assessment Service; SIDIAP-H: Information System for Research in Primary Care—Hospitalization Linked Data; VA-OMOP: Department of Veterans Affairs. CKD: chronic kidney disease; COPD: chronic obstructive pulmonary disease; GERD: gastro-oesophageal reflux disease; NA: not available; PPI: proton pump inhibitor.

A total of 48 418 patients (46 721 from USA, 884 from Spain and 813 from South Korea) with autoimmune diseases were hospitalized with COVID-19 (Table 2). Patients were mainly female in CUIMC (54.8%), HIRA (63.5%), IQVIA Open Claims (54.8%), Optum EHR (59.5%), about equal proportion in SIDIAP-H (49.0%), but were predominantly male in VA-OMOP (93.2%). Majority of cases were aged ≥50 years. Among these patients with autoimmune diseases who were hospitalized with COVID-19, Type 1 diabetes was the most common autoimmune condition in the US databases (4.8–7.5%) whereas RA was most prevalent in HIRA (18.9%) and psoriasis in SIDIAP-H (26.4%). The most prevalent comorbidities were hypertension (45.5–93.2%), heart disease (29.0–83.8%), Type 2 diabetes (30.8–74.3%) and hyperlipidaemia (31.9–64.5%). The most frequently prescribed medications in the year prior to hospitalization across all databases were systemic antibiotics (52.4–84.0%), drugs used for GERD (47.9–80.6%) and NSAIDs (31.4–81.5%). The list of patient characteristics is presented in Tables 1 and 2. A full list of the conditions that make up the prevalent autoimmune diseases is presented in Supplementary Tables S1 and S2, available at *Rheumatology* online. A complete list of patient characteristics can be found in the aforementioned interactive web application.

**Table 2:** Baseline characteristics of study participants hospitalized with COVID-19 and who had prevalent autoimmune disease, stratified by data source

Covariate	CUIMC (USA) (*n* = 557)	HIRA (South Korea) (*n* = 813)	IQVIA Open Claims (USA) (*n* = 39 900)	Optum EHR (USA) (*n* = 3112)	SIDIAP-H (Spain) (*n* = 884)	VA-OMOP (USA) (*n* = 3152)
Female	54.8	63.5	54.8	59.5	49.0	6.8
Age group						
≤19	<3.6	0.7	0.8	0.8	<0.6	0.2
20–29	2.0	8.8	1.2	3.0	0.6	0.2
30–39	2.9	4.5	2.9	6.9	2.5	1.4
40–49	5.2	9.9	6.0	10.5	8.0	3.3
50–59	10.7	26.7	14.4	18.1	14.3	10.9
60–69	20.5	21.9	23.8	25.0	17.7	25.3
70–79	23.4	15.2	25.5	19.3	25.3	39.3
80–89	23.9	9.9	25.4	16.2	24.4	13.4
90–99	8.5	1.8	0.0	0.0	6.7	6.0
Autoimmune disease in the year prior to index date^a^						
Type 1 diabetes mellitus	4.8	1.5	7.5	7.5	4.4	5.3
RA	4.8	18.9	4.9	8.8	5.4	4.0
Psoriasis	1.4	8.2	2.7	5.4	26.4	4.4
PsA	0.0	0.7	0.6	1.7	2.5	0.9
Multiple sclerosis	1.1	<0.6	2.1	3.7	2.1	1.6
SLE	3.2	1.7	1.9	4.3	2.6	0.9
Hashimoto thyroiditis	0.0	0.0	0.0	0.9	0.0	0.0
Myasthenia gravis	<0.9	<0.6	0.5	0.8	1.5	0.7
Vasculitis	3.4	14.4	4.4	7.7	20.8	4.4
Pernicious anaemia	0.0	0.0	0.0	0.4	0.0	0.0
Coeliac disease	<0.9	<0.6	0.3	0.9	1.2	0.4
Scleroderma	<0.9	<0.6	0.2	0.4	0.9	<0.2
Sarcoidosis	3.4	0.0	1.2	1.9	1.2	2.1
Ulcerative colitis	<0.9	<0.6	1.3	2.2	2.8	1.6
Crohn's disease	1.1	<0.6	1.0	2.4	2.4	1.2
Comorbidities in the year prior to index date						
Hyperlipidaemia	45.8	56.5	44.9	49.2	31.9	64.5
Asthma	29.3	28.0	22.1	20.4	7.8	12.5
CKD	50.4	14.0	49.6	42.0	25.8	52.7
COPD	28.7	3.4	30.8	26.3	40.7	51.9
Dementia	23.2	12.2	21.5	8.9	6.8	23.1
Heart disease	83.8	29.0	81.7	62.7	47.7	81.9
HIV	2.7	NA	2.4	1.1	0.7	2.2
Hypertension	91.4	45.5	91.6	75.7	58.9	93.2
Cancer	38.4	8.5	29.0	29.4	22.6	37.9
Obesity	67.7	NA	48.0	67.0	57.5	64.0
Type 2 diabetes mellitus	70.0	46.1	74.1	50.6	30.8	74.3
Cerebrovascular disease	10.8	7.6	11.2	6.8	4.1	9.5
Chronic liver disease	4.1	10.2	2.9	3.4	3.3	9.7
Pregnancy	2.0	2.0	0.7	2.7	0.6	NA
Venous thromboembolism	11.3	3.4	8.7	8.8	14.4	9.3
Drug utilization in the year prior to index date						
Agents acting on the renin–angiotensin system	37.0	29.4	36.7	38.3	43.9	51.8
Antibacterials	52.4	84.0	55.0	57.6	59.4	64.4
Antidepressants	23.7	21.2	25.0	33.6	31.3	47.0
Antineoplastic and immunomodulating agents	23.0	10.0	15.4	23.3	18.3	19.3
Antithrombotics	55.3	46.9	32.8	55.5	45.5	69.3
Beta blockers	41.8	17.1	35.1	40.3	26.1	54.9
Calcium channel blockers	37.0	25.3	28.1	29.4	21.2	41.4
Corticosteroids	37.7	72.3	39.5	48.5	48.6	46.2
Diuretics	41.8	17.3	33.4	38.9	41.4	47.9
Drugs for obstructive airway diseases	35.7	30.1	39.8	46.9	31.9	58.1
GERD	47.9	80.6	54.1	49.3	61.2	77.8
PPI	36.6	50.3	28.0	40.5	60.3	48.4
Lipid modifying agents	49.6	34.6	42.5	47.4	39.5	71.4
NSAID	33.0	77.6	51.9	35.6	31.4	81.5
Opioids	30.7	82.0	28.0	41.4	28.2	37.7

Figures are presented in percentages; the figures preceded with < denote less than five people in that category. Age groups are collapsed from 5-year age bands; percentages are not summed if one of the age categories had less than five people.

a These are not mutually exclusive, and classification is based on recent (1 year prior) records.

CUIMC: Columbia University Irving Medical Center; HIRA: Health Insurance Review and Assessment Service; SIDIAP-H: Information System for Research in Primary Care—Hospitalization Linked Data; VA-OMOP: Department of Veterans Affairs. CKD: chronic kidney disease; COPD: chronic obstructive pulmonary disease; GERD: gastro-oesophageal reflux disease; NA: not available; PPI: proton pump inhibitor.

**Fig. 1:**
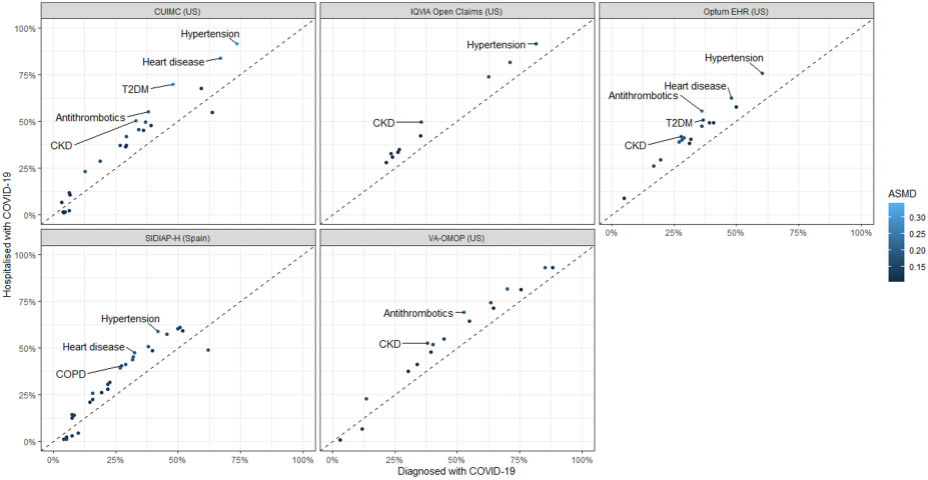
Prevalence of patient characteristics in the patients with prevalent autoimmune diseases who were diagnosed with COVID-19 compared with those hospitalized with COVID-19 This scatterplot includes patient characteristics with absolute standardized mean difference (ASMD) ≥0.1. The patient characteristics with ASMD >0.2 are labelled in the scatterplot. HIRA was not included in the scatterplot because of the significant overlap between diagnosed (*n* = 815) and hospitalized (*n* = 813) patients. CKD: chronic kidney disease; COPD: chronic obstructive pulmonary disease; T2DM: Type 2 diabetes mellitus

In patients with prevalent autoimmune diseases hospitalised with COVID-19, the prevalence of hypertension (ASMD = 0.18–0.34), chronic kidney disease (ASMD = 0.17–0.25), heart disease (ASMD = 0.18–0.28), Type 2 diabetes (ASMD = 0.15–0.32), chronic obstructive pulmonary disease (COPD) (ASMD = 0.11–0.20) and use of antithrombotics (ASMD = 0.15–0.28) were higher as compared with the larger group of such patients diagnosed with COVID-19 (Fig. 1).

**Fig. 2:**
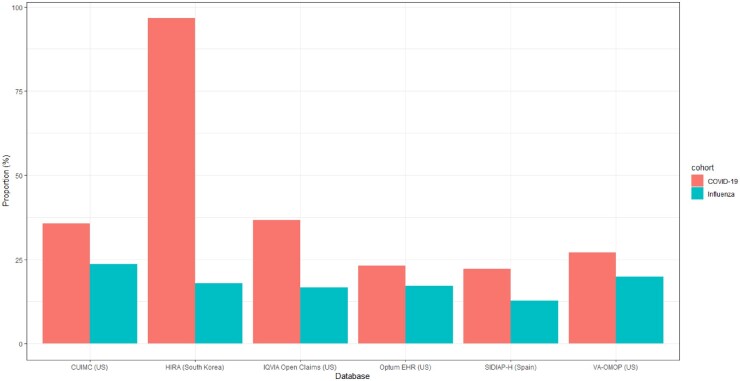
Hospitalization in patients with prevalent autoimmune diseases in the 30-day period following a diagnosis of COVID-19 *vs* influenza

We included 395 784 patients with prevalent autoimmune diseases (392 797 from USA, 2419 from Spain and 568 from South Korea) diagnosed with influenza to compare the proportion of hospitalization episodes. The proportion of hospitalization episodes was higher in the cohort diagnosed with COVID-19 as compared with influenza [35.7% *vs* 23.6% (CUIMC), 96.6% vs 18.0% (HIRA), 36.7% *vs* 16.6% (IQVIA Open Claims), 23.1% *vs* 17.2% (Optum EHR), 22.2% *vs* 12.8% (SIDIAP-H), 27.1% vs 19.9% (VA-OMOP)] (Fig. 2).

**Fig. 3:**
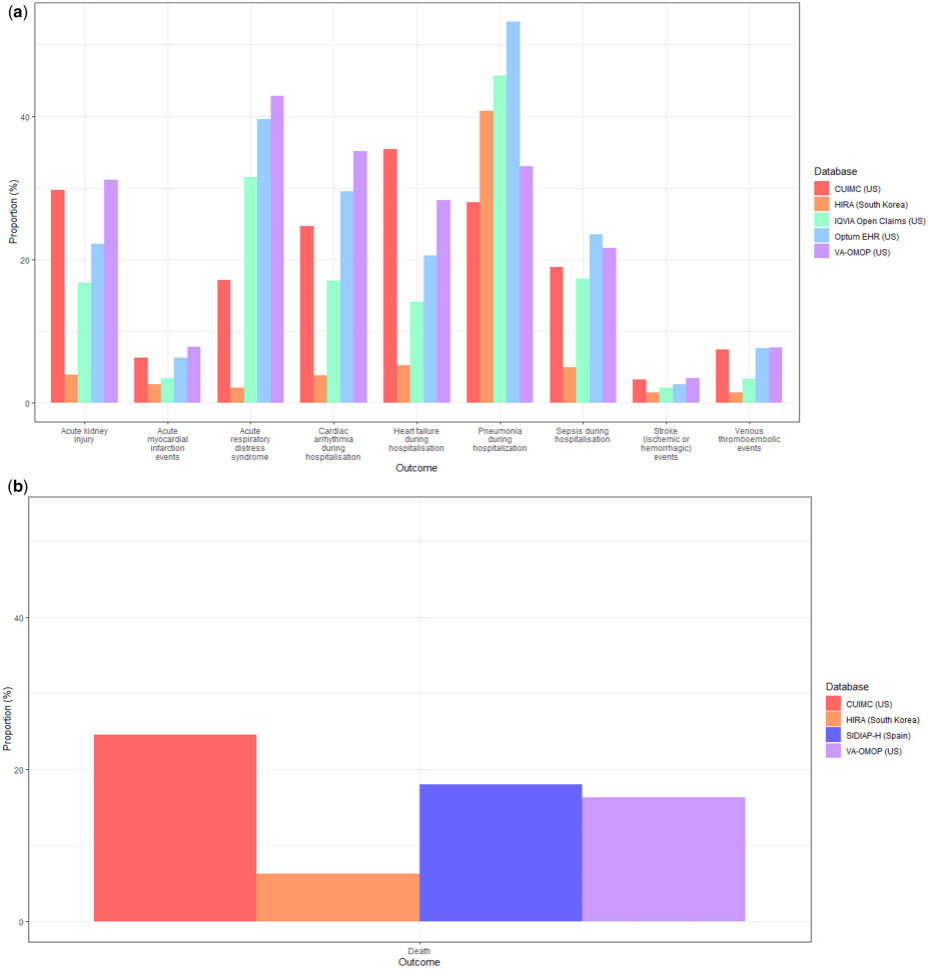
(a) Severe outcomes in 30 days post hospital admission with COVID-19 in patients with prevalent autoimmune diseases, stratified by database Hospitalization outcomes data was not available in SIDIAP-H. (**b**) Mortality in 30 days post hospital admission with COVID-19 in patients with prevalent autoimmune diseases, stratified by database.

At 30 days post hospitalization, the most frequent severe outcomes were related to the respiratory system, such as ARDS (2.1–42.8%), and pneumonia (12.6–53.2%) (Fig. 3a). Acute kidney injury was the second most common complication, occurring in 9.9–31.1% of patients in US databases, and in 2.8% in HIRA. Cardiac complications were also frequent, including arrhythmia in 3.8–35.1% of patients, heart failure (3.9–24.5%) and acute myocardial infarction (2.4–6.3%). Sepsis occurred during hospitalization in 4.7–23.5% of patients. Ischaemic or haemorrhagic stroke was recorded in 1.4–3.4% of patients, whereas venous thromboembolic events (VTEs) were recorded in 1.4–7.7% of patients across the databases. Mortality as a proportion of those hospitalized was generally higher in the USA and Spain (16.3–24.6%) *vs* South Korea (6.3%) (Fig. 3b).

**Fig. 4:**
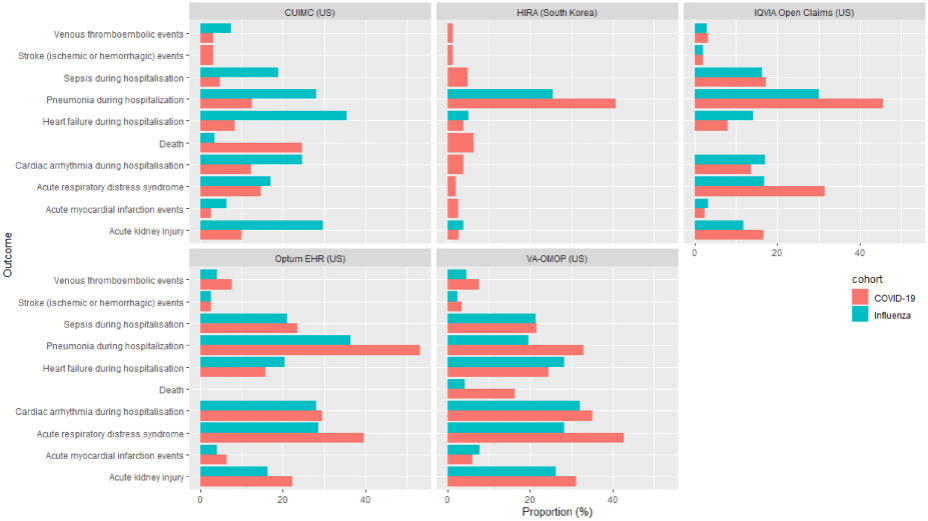
Comparison of outcomes in patients with prevalent autoimmune conditions hospitalized with COVID-19 *vs* influenza Outcomes were omitted from the graph if there were less than five people experiencing the event or the data was unavailable in the respective databases. CPRD: Clinical Practice Research Datalink; CUIMC: Columbia University Irving Medical Center; DA Germany: IQVIA disease analyser Germany; DCMC: Daegu Catholic University Medical Center; HIRA: Health Insurance Review and Assessment Service; LPD France: IQVIA Longitudinal Patient Data France; NFHCRD: Nanfang Hospital COVID-19 Research Database; IPCI: Integrated Primary Care Information; SIDIAP: Information System for Research in Primary Care; SIDIAP-H: SIDIAP- Hospitalisation Linked Data; TRDW: Tufts Research Data Warehouse, VA-OMOP: Department of Veterans Affairs.

Compared with 70 660 hospitalized individuals (70 184 from USA, 323 from Spain and 153 from South Korea) with influenza in previous years, patients hospitalized with COVID-19 were more likely to have higher respiratory complications such as ARDS (14.7–42.8% *vs* 16.9–28.7%) and pneumonia (12.6–53.2% *vs* 19.5–36.3%), and had a higher mortality (6.3–24.6% *vs* 2.2–4.4%) (Fig. 4) (Supplementary Table S3, available at *Rheumatology* online).

## Discussion

This study represents the hitherto first use of routinely collected health data across the USA, Spain and South Korea to characterize hospitalized COVID-19 patients with prevalent autoimmune diseases. To our knowledge, this is the largest multinational observational study to characterize a cohort of patients with prevalent autoimmune diseases diagnosed/hospitalized with COVID-19 and detail their post-hospitalization outcomes, during the first 6 months of the pandemic. We found that diagnosed autoimmune patients were predominantly female, aged >50 years and had pre-existing comorbidities. Hospitalized autoimmune patients had similar characteristics to those diagnosed but were older and had a higher proportion of pre-existing comorbidities. As compared with patients hospitalized with influenza, more patients infected with COVID-19 died within 30 days of hospitalization. COVID-19 patients also experienced respiratory and cardiac complications during hospitalization.

The patients in our study were predominantly female, except for the VA-OMOP database, of which the majority were male military veterans. This was consistent with the proportion of females across studies of COVID-19 in patients with autoimmune conditions in Spain (59%) [12] and in COVID-19 patients in the Global Rheumatology Alliance (GRA) physician-reported registry (67%) [19]. This is likely due to females having a higher prevalence of most autoimmune diseases, but contrasts with reports of overall COVID-19 patients who were otherwise majority male [16, 28]. A recent meta-analysis has also shown that males had higher in-hospital mortality [29]. The hospitalized patients in our study were mostly aged 65 years and above, with South Korea having more patients in the age group of 50–64 years old. Advanced age has been reported as a poor prognostic factor for COVID-19 [15, 29]. The sociodemographic profile of the patients in VA-OMOP being mostly male and older could be associated with the higher frequency of severe outcomes in that data source. The most prevalent comorbidities in our study were hypertension, heart disease and Type 2 diabetes. This was similarly observed in the GRA registry [19]. These comorbidities were also associated with disease severity and mortality in a meta-analysis involving 12 149 general COVID-19 patients from 15 countries [29].

Our study described post-hospitalization complications in COVID-19 patients with prevalent autoimmune diseases. The most frequent severe outcomes in our study were ARDS, pneumonia and cardiac injury. In the aforementioned meta-analysis [29], the most frequently reported complications associated with COVID-19 were pneumonia, respiratory failure, acute cardiac injury and ARDS; which corroborates our findings regarding the frequency of outcomes. Cardiac injury was also independently associated with in-hospital mortality in a study conducted in Wuhan [30]. The researchers hypothesized that cardiac injury may be precipitated by acute inflammatory response as a result of COVID-infection superimposed on pre-existing cardiovascular disease. In comparison with patients hospitalized with influenza, COVID-19 patients generally had higher proportion of severe outcomes, especially respiratory complications such as ARDS and pneumonia. This phenomenon was also observed in a study conducted in a large tertiary care hospital in the USA, where patients hospitalized with COVID-19 required more mechanical ventilation and had higher mortality than patients with influenza, despite presenting with less pre-existing conditions [31]. Our study showed that up to 8% of hospitalized patients with COVID-19 and prevalent autoimmune diseases suffered VTE and the incidence of VTE is higher in hospitalized COVID-19 patients *vs* influenza patients in most of the databases. There are extensive evidence demonstrating increased risk of thromboembolism among patients with autoimmune disease, where the mechanism is hypothesized to be a relationship between inflammation and the coagulation pathway [6–8]. The underlying mechanism associated between COVID-19 and intravascular thrombosis has not been fully elucidated. Suggested explanations include the activation of the thrombo-inflammation pathway, endothelial injury and microangiopathy [32]. As COVID-19 and autoimmune disease has been associated with higher risk of VTE, it is plausible that the confluence of both these conditions may heighten this risk. Using a multicentre EHR network, D’Silva *et al.* [33] found that patients with systemic autoimmune rheumatic diseases diagnosed with COVID-19 had higher risks of VTE as compared with matched patients, and this increased risk of VTE was not mediated by comorbidities.

There was a high prevalence of comorbidities among the patients in our study. According to a meta-analysis [34], the presence of comorbidities such as hypertension, diabetes and obesity among patients with autoimmune diseases was associated with higher rates of hospitalization, ventilation and death due to COVID-19. In a large study using primary care records in England, patients with autoimmune disease had an increased risk of COVID-19 related death after adjustment for various comorbidities such as hypertension, diabetes, cancer, heart disease and respiratory disease [35]. In other patient populations, such as those with obesity, patients with COVID-19 had higher mortality and requirement of intensive services as compared with similar patients with seasonal influenza, despite presenting with fewer comorbidities [36]. In pregnant women, there was a higher frequency of caesarean section and preterm deliveries, as well as poorer outcomes (pneumonia, ARDS, sepsis, acute kidney injury and cardiovascular and thromboembolic events) in those diagnosed with COVID-19 in comparison with seasonal influenza [37]. Like the other databases, CUIMC showed higher mortality in hospitalized patients with COVID-19 than those with influenza, but it showed lower complications in patients hospitalized with COVID-19 than influenza. A possible explanation is that patients hospitalized with influenza had higher incidence of co-morbidities like COPD and Type 2 diabetes, which was also found in a previous study [38], or that data were not well captured during the height of the pandemic.

### Study limitations

Although our study found a greater proportion of hospitalization with COVID-19 as compared with influenza, the hospitalization rate may not directly reflect the severity of prognosis in COVID-19. As a novel coronavirus, the higher hospitalization rate could also be contributed by quarantine measures in the hospital after diagnosis or monitoring of patients receiving investigative treatments repurposed for COVID-19. Hence, we have further provided more information regarding severe outcomes within 30 days of hospitalization. COVID-19 cases may be poorly recognized due to shortages in testing capabilities, but this is to some extent mitigated in our study by also including hospitalized patients with a clinical COVID-19 diagnosis. However, even untested hospitalized patients could have been missed if hospitals were understaffed and clinicians did not have time to input proper codes. A known limitation of using routinely collected data is that medical conditions may be misclassified due to erroneous entries or underestimated as they were defined based on the presence of diagnostic or procedural codes, with the absence of records indicative of absence of disease. In particular for healthcare data in the USA, the capturing of codes is largely incentivized by reimbursement from insurance companies. This factor could permit miscoding of Type 2 diabetes as Type 1 and could have enriched the autoimmune disease cohort with Type 2 diabetes patients who might not have autoimmune disease. With the use of claims databases, there may be a discrepancy between the diagnosis recorded and the actual health condition of a patient. However, this is mitigated in more severe conditions and inpatient settings [23]. In the initial stage of the pandemic, the lack of clinical guidance combined with the lack of access to widespread testing means that only more severe patients were seen in healthcare settings. The capture of mortality data is subject to differences by database. For example, data on inpatient deaths are recorded in a hospital EHR but deaths after discharge from hospital will not be captured in such a data source. For data sources linked to primary care, outpatient death events are typically imported into a given database from a national or local death register. It is likely that mortality rates were underestimated in our study. Nevertheless, the consistency of our findings across different healthcare settings in different countries lends credence to our results.

## Conclusions

Patients with autoimmune diseases had high rates of respiratory complications and 30-day mortality following a hospitalization with COVID-19. Compared with influenza, COVID-19 is a more severe disease, leading to more complications and higher mortality. Future studies should investigate predictors of poor outcomes in COVID-19 patients with autoimmune diseases.

## Supplementary Material

keab250_Supplementary_Data
