# Supplementary material for: COVID-19 in patients with autoimmune diseases: characteristics and outcomes in a multinational network of cohorts across three countries
Source: Rheumatology (Oxford). 2021 Mar 16;60(SI):SI37–50. doi: 10.1093/rheumatology/keab250 (PMC7989171; doi:10.1093/rheumatology/keab250)
Supplement: keab250_Supplementary_Data [file keab250_supplementary_data.zip › Supplementary Appendix.docx]

**Supplementary Appendix 1**

| **Data source** | **Source population** | **Sample size** | **Data type** | **Longitudinal history** |
| --- | --- | --- | --- | --- |
| Columbia University Irving Medical Center (CUIMC) | Patients of the Columbia University Irving Medical Center (New York City, USA) | ≈ 6 million | The clinical data warehouse of New York-Presbyterian Hospital/Columbia University Irving Medical Center, New York, NY, based on its current and previous electronic health record systems, with data spanning over 30 years and including over 6 million patients | 1989 (1978 for diagnoses) to June 2020 |
| Health Insurance and Review Assessment (HIRA) | All citizens in South Korea | ≈ 50 million | Administrative fee-for-service claims data collected for healthcare reimbursement, including healthcare services such as treatments, pharmaceuticals, procedures, and diagnoses. | 5-years of available look-back (data older than 5-years is deleted from the database) |
| IQVIA Open Claims | USA | >300 million | A United States database of open, pre-adjudicated medical and pharmacy claims. Data are reported at anonymised patient level collected from office-based physicians and specialists via office management software and clearinghouse switch sources for the purpose of reimbursement. A subset of medical claims data have adjudicated claims. | January 2013 to May 2020 |
| Optum EHR | USA | ≈ 1.4 million | Optum® de-identified COVID-19 Electronic Health Record dataset represents Optum’s Electronic Health Record data a medical records database for patients receiving a COVID-19 diagnosis record or lab test for SARS-CoV-2. The medical record data includes clinical information, inclusive of prescriptions as prescribed and administered, lab results, vital signs, body measurements, diagnoses, procedures | January 2007 to June 2020 |
| The Information System for Research in Primary Care (SIDIAP-H) | General population in Catalonia, Spain | ≈ 2 million | SIDIAP is a primary care records database that covers approximately 7 million people, equivalent to an 80% of the population of Catalonia, North-East Spain. The SIDIAP-H subset of the database includes around 2 million people out of the total 7 million in SIDIAP that are registered in primary care practices with linked hospital inpatient data (up to 2018 only) available as obtained from the Catalan Institute of Health hospitals. Healthcare is universal and tax-payer funded in the region, and primary care physicians are gatekeepers for all care and responsible for repeat prescriptions. | 2006 to June 2020 |
| United States Department of Veterans Affairs (VA-OMOP) | Patients of the Veterans Affairs in the United States | ≈ 9 million | VA OMOP data reflects the national Department of Veterans Affairs health care system, which is the largest integrated provider of medical and mental health services in the United States. Care is provided at 170 VA Medical Centers and 1,063 outpatient sites serving more than 9 million enrolled Veterans each year. | 2000 to August 2020 |
